# Early decline in serum phospho-CSE1L levels in vemurafenib/sunitinib-treated melanoma and sorafenib/lapatinib-treated colorectal tumor xenografts

**DOI:** 10.1186/s12967-015-0553-6

**Published:** 2015-06-13

**Authors:** Woan-Ruoh Lee, Shing-Chuan Shen, Yi-Hsien Shih, Chia-Lun Chou, Jonathan Te-Peng Tseng, Szu-Ying Chin, Kao-Hui Liu, Yen-Chou Chen, Ming-Chung Jiang

**Affiliations:** Department of Dermatology, Shuang Ho Hospital, Taipei Medical University, New Taipei, Taiwan; Department of Dermatology, School of Medicine, College of Medicine, Taipei Medical University, No. 252 Wu-Hsing St., Taipei, 11031 Taiwan; Graduate Institute of Medical Sciences, College of Medicine, Taipei Medical University, Taipei, Taiwan

**Keywords:** ERK1/2, Monitoring, Phospho-CSE1L, Serum, Targeted drug, Targeted therapy

## Abstract

**Background:**

Although targeted therapies have improved the clinical outcomes of cancer treatment, tumors resistance to targeted drug are often detected too late and cause mortality. CSE1L is secreted from tumor and its phosphorylation is regulated by ERK1/2. ERK1/2 is located downstream of various growth factor receptors and kinases, the targets of most targeted drugs. Serum phospho-CSE1L may be a marker for monitoring the efficacy of targeted therapy.

**Methods:**

We used mice tumor xenograft model to study the assay of serum phosphorylated CSE1L for early detecting the efficacy of targeted drugs. The phosphorylation status of CSE1L in vemurafenib and sorafenib treated tumor cells were assayed by immunoblotting with antibody against phosphorylated CSE1L.

**Results:**

Ras activation increased phospho-CSE1L expression in B16F10 melanoma cells. Vemurafenib and sorafenib treatment did not significantly reduce the total CSE1L levels; however, they inhibited ERK1/2 and CSE1L phosphorylation in A375 melanoma cells and HT-29 colorectal cancer cells. In the melanoma xenograft model, serum phospho-CSE1L level declined 5 days after vemurafenib/sunitinib treatment and 3 days after sorafenib/lapatinib treatment in the HT-29 colon cancer xenograft model. Vemurafenib/sunitinib and sorafenib/lapatinib treatments resulted in tumor regression.

**Conclusions:**

Our results indicated that serum phospho-CSE1L is useful for early detecting the efficacy of targeted therapy in initial treatment and for monitoring emerging secondary drug resistance to facilitate timely therapeutic decision making.

## Background

Compared with empirical chemotherapy, targeted therapy yields substantially improved clinical outcomes in cancer treatment. However, the efficacy of targeted therapies in cancer patients is often limited by the emergence of resistance. For example, the response rate was approximately 50% for vemurafenib in melanoma with B-Raf V600E mutation [1]. Moreover, numerous patients eventually developed resistance to the drugs within 1 year of treatment, despite successful initial treatment [2]. Another example is that the response rate of Cetuximab monotherapy was 11%, and was 23% in combination with irinotecan for colorectal cancer treatment [3]. The progression of a tumor may be rapid, and tumor resistance to a targeted drug is often detected too late. Therefore, it is essential to assess the efficacy of targeted therapy in initial treatment and monitor the development of resistance to the targeted drug after a successful initial treatment.

The Ras-Raf-MAPK-ERK signaling cascade as well as its upstream growth factor receptors are frequently activated in cancer and are the targets of most targeted drugs [4–7]. For example, vemurafenib (PLX4032, RG7204) is a potent inhibitor of B-Raf V600E in melanoma treatment [8]. Sorafenib (BAY 43-9006) is a multi-kinase inhibitor of Raf-1, B-Raf, and vascular endothelial cell growth factor receptor-2 (VEGFR-2) [9]. Sorafenib inhibits the phosphorylation of mitogen-activated protein kinase (MAPK) and extracellular signal-regulated kinase (ERK1/2) in various cancer cell lines and tumor xenografts [10–12]. Sunitinib malate (Sutent, SU11248; Pfizer Inc.) is a multi-targeted receptor tyrosine kinase (RTK) inhibitor of VEGFRs, platelet-derived growth factor receptors (PDGFRs), SCF receptor (c-kit), and FLT3 [13–15]. Lapatinib, a potent human epidermal growth factor receptor 2 (HER2) and epidermal growth factor receptor (EGFR) inhibitor, is an oral drug for breast cancer and other solid tumors [16, 17]. ERK1/2 is located downstream of the Raf/MEK/ERK (MAPK) signal transduction cascade, at which cancer signaling from various upstream growth factor receptors, such as ErbB/HER, VEGFR, PDGFR, c-kit, and hepatocyte growth factor receptor (Met), and from Ras, Raf, and MAPK/ERK kinase (MEK) signaling converge [4–7, 18–23]. All of these receptors and kinases are potential targets for drugs designed for cancer therapy. Therefore, the activity of ERK1/2 in targeted drug-treated tumor can indicate the efficacy of the drug. It is difficult to obtain tumor tissue through biopsy to assay the ERK1/2 activity in a tumor in clinical setting. Biomarkers that are linked with ERK1/2 signaling and are secreted from tumor may be excellent markers for assessing tumor responses to a targeted drug, and thus can be used in early detection of the efficacy of a targeted therapy.

The chromosome segregation 1-like (CSE1L) protein is the human homologue of CSE1, the yeast chromosome segregation protein [24]. Pathological studies have shown that CSE1L is highly expressed in most cancer types, and its expression is associated with advanced cancer stage and poor outcome in cancer patients [25–27]. We have previously reported that CSE1L is a secretory protein present in the sera of cancer patients [28–30]. Our previous studies have shown that CSE1L is a phosphorylated protein, and its phosphorylation is regulated by Ras-ERK signaling [31, 32]. We studied serum phospho-CSE1L for assaying the efficacy of targeted therapy using mice melanoma and colorectal tumor xenograft models and drugs including vemurafenib, sorafenib, sunitinib, and lapatinib. Here, we report that serum phospho-CSE1L can be used to monitor the efficacy of targeted drugs as early as 3 days after drug administration. The results suggested that serum phospho-CSE1L has clinical application in early detecting the development of resistance to targeted drugs to improve the cure rate of cancer.

## Methods

### Antibodies, targeted drugs, and reagents

The antibodies used in the experiment were anti-p21/ras (EP1125Y) (Epitomics, Burlingame, CA, USA); anti-CSE1L (3D8) and anti-phospho-ERK1/2 (phospho T202/204, G15-B) (Abnova, Taipei, Taiwan); anti-β-actin (Ab-5) (Lab Vision, Fremont, CA, USA); anti-CSE1L (24) and anti-CSE1L (H2) (Santa Cruz Biotechnology, Santa Cruz, CA, USA). PD98059 and other reagents were obtained from Sigma (St. Louis, MO, USA).

The targeted drugs used in the experiment were vemurafenib (Selleck South Loop West, TX, USA), sorafenib tosylate (Santa Cruz Biotechnology), sunitinib (Pfizer Inc., New York, NY, USA), and lapatinib ditosylate (Tykerb) (GlaxoSmithKline plc, Brentford, England).

### Cells and cell cultures

A375 human melanoma cells were obtained from the American Type Culture Collection (Manassas, VA, USA). NIH 3T3 mouse embryo fibroblast cells, human foreskin fibroblast cells, HT-29 colorectal cancer cells, and B16F10 mouse melanoma cells were maintained in our laboratory previously [31–33]. Cells were cultured in Dulbecco’s modified Eagle’s medium (DMEM) supplemented with 10% heat-inactivated fetal bovine serum (FBS), 100 units/mL of penicillin, 100 mg/mL of streptomycin, and 2 mmol/L of glutamate at 37°C in a humidified 5% CO_2_ atmosphere. B16F10 melanoma cells expressing v-H-Ras expression vectors (i.e. the B16-Ras cells), CSE1L expression vectors (i.e. the B16-CSE1L cells), and the control vectors (i.e. the B16-dEV cells) were established previously [31].

### Production of antibodies specific to phosphorylated CSE1L

Phosphopeptide, L**T**^**p**^E**Y**^**p**^LKKTLDPDPAC (**T**^**p**^ denotes phospho-threonine and **Y**^**p**^ denotes phospho-tyrosine), and non-phosphopeptide, LTEYLKKTLDPDPAC, were synthesized using the solid-phase method. The phosphorylated peptides were conjugated through the N-terminal cysteine thiol to keyhole limpet hemocyanin (KLH). New Zealand rabbits were immunized with the peptides five times. The immunized serum was collected a week after the final immunization. IgG fractions were purified using a protein G column (Amersham Pharmacia Biotech, Uppsala, Sweden). The antibodies were purified using a phosphorylated peptide affinity column followed by non-phosphopeptide cross-adsorption to remove non-phospho-specific antibodies. The titer and the specificity of the antibodies were tested by ELISA and immunoblotting.

### Immunoblotting

All cell lysates were prepared using lysis buffer containing phosphatase inhibitors unless otherwise indicated. Cells were washed with phosphate-buffered saline (PBS) and lysed in ice-cold radioimmunoprecipitation assay (RIPA) buffer (25 mM Tris–HCl [pH 7.2], 0.1% SDS, 0.1% Triton X-100, 1% sodium deoxycholate, 150 mM NaCl, 1 mM EDTA, 5 mM sodium orthovanadate, 1 mM phenylmethylsulfonyl fluoride, 10 μg/mL of aprotinin, and 5 μg/mL of leupeptin) containing phosphatase inhibitors (25 mM β-glycerophosphate and 5 mM sodium fluoride). The protein concentrations were determined using a BCA protein assay kit (Pierce, Rockford, IL, USA). Protein samples (50 μg each) were loaded onto an SDS–polyacrylamide gel. Proteins were transferred to nitrocellulose membranes (Amersham Pharmacia, Little Chalfont, Buckinghamshire, UK). The membrane was incubated with blocking buffer (1% bovine serum albumin [BSA], 50 mM Tris–HCl [pH 7.6], 150 mM NaCl, and 0.1% Tween-20) for 1 h. The blots were reacted with primary antibodies at 4°C and incubated overnight; subsequently, they were incubated with secondary antibodies conjugated to horseradish peroxidase (HRP) for 1 h. The protein levels were detected by enhanced chemiluminescence by using a Forte Western HRP substrate (Millipore, Billerica, MA, USA).

### Immunofluorescence

Cells grown on coverslips (12 × 12 mm) were cytospun at 1,000 rpm for 10 min. The cells were washed with PBS, fixed with 4% paraformaldehyde, permeabilized with methanol, and blocked with PBS containing 0.1% BSA. The cells were then incubated with primary antibodies for 1 h, washed with PBS three times, and incubated with goat anti-mouse IgG secondary antibodies coupled to Alexa Fluor 488. Subsequently, the cells were examined using a Zeiss Axiovert 200 M inverted fluorescence microscope (Carl Zeiss, Jena, Germany). Experiments were performed on duplicate coverslips, and ten random fields were photographed per coverslip.

### Protein phosphatase treatment

For protein phosphatase treatment, cells were washed with PBS and lysed in phosphatase-inhibitor-depleted RIPA buffer (25 mM Tris–HCl [pH 7.2], 0.1% SDS, 0.1% Triton X-100, 1% sodium deoxycholate, 150 mM NaCl, 1 mM phenylmethylsulfonyl fluoride, 10 μg/mL of aprotinin, and 5 μg/mL of leupeptin) at 4°C. The cell lysate was incubated with protein phosphatase reaction buffer containing 50 mM HEPES (pH 7.5), 10 mM NaCl, 2 mM DTT, 0.01% Brij 35, 1 mM MnCl_2_, and 4,000 units of lambda protein phosphatase (New England Biolabs, Ipswich, MA, USA) in a total volume of 100 μL at 30°C for 1 h.

### DNA fragmentation assay

Cells cultured in cultured medium containing 10% FBS were treated with vemurafenib for 96 h. The detached cells in media and attached cells harvested with trypsin–EDTA digestion were combined and centrifuged at 1,000*g* for l0 min. The cell pellets were incubated in lysis buffer (50 mM Tris–HCl [pH 8.0], 10 mM EDTA, 0.5% sarkosyl, 0.5 mg/mL of proteinase K) at 50°C for 1.5 h and then treated with 0.5 μg/mL of RNase A for 30 min. The DNA was extracted using phenol–chloroform-isoamyl alcohol and analyzed by gel electrophoresis (1.8% agarose gel) with ethidium bromide staining. The amount of DNA loaded in each well was 20 μg.

### Animal targeted therapy model

Male NOD SCID mice (8 weeks of age; National Laboratory Animal Center, Taipei, Taiwan) were housed in a clean room and maintained in sterilized filter-topped cages equipped with high-efficiency particulate air (HEPA) filter-ventilated racks. The mice received sterile rodent chow and water ad libitum. Each mouse was subcutaneously inoculated with viable cancer cells (1 × 10^4^ cells in 100 μL of PBS/mouse) in the dorsal skin by using a 26-gauge needle.

Vemurafenib (75 mg/kg) and sunitinib (20 mg/kg) were dissolved in dimethyl sulfoxide (DMSO) and suspended in an aqueous vehicle solution containing 2% hydroxypropyl cellulose (Sigma) in PBS; this mixture was adjusted to pH 4 by using dilute HCl. Sorafenib (80 mg/kg) and lapatinib (60 mg/kg) were suspended in an aqueous vehicle solution containing 2% hydroxypropyl cellulose in PBS, and the mixture was adjusted to pH 4 by using dilute HCl. For the control groups, lapatinib (1 mg/kg) was suspended in an aqueous vehicle containing 2% hydroxypropyl cellulose in PBS, and the mixture was adjusted to pH 4 by using dilute HCl. When the tumors attained a volume of approximately 100–150 mm^3^, each mouse was labeled using a mouse ear punch. Each mouse was then weighed, and blood (approximately 200 μL) was extracted from the facial vein by using a lancet. The mice were divided into two groups, and each group had mice bearing tumors of similar sizes. Beginning on the next day, the mice were fed with the targeted drugs (in a volume of 100 μL) through oral gavage by using a feeding needle (gavage needle) daily for 10 days. Mice were fed with vemurafenib and sunitinib (for A375 melanoma cells-injected mice), sorafenib and lapatinib (for HT-29 colorectal cancer cells-injected mice), or lapatinib (1 mg/kg) (for the control groups). Vemurafenib may work in melanoma patients whose tumor has a B-Raf V600E mutation, and A375 melanoma cells harbor the mutation [34]. Lapatinib was reported to be unable to prevent the growth of A375 melanoma cells [35]. HT-29 cancer cells carry B-Raf mutation [36]. K-Ras/B-Raf mutations are associated with resistance to lapatinib, and the HT-29 colorectal cancer cell line was reported to be resistant to lapatinib [37]. The group of mice injected with HT-29 colorectal cancer cells consisted of 15 mice, the group of mice injected with A375 melanoma cells consisted of 10 mice, and each control group consisted of five mice. The tumor size of each mouse was measured using a size caliper every 2 days. Blood (about 200 μL) was collected from each mouse injected with HT-29 cells 3 days post the first day of drug administration, and 5 days post the first day of drug administration for mouse injected with A375 melanoma cells. Serum samples were collected by allowing blood to stand at room temperature for a minimum of 30 min to allow clots to form. The blood samples were centrifuged at 2,000*g* for 10 min, and the sera in supernatants were subsequently harvested and stored at −80°C. The tumor volume (cm^3^) was calculated using the following formula: W^2^ × L × 0.5, where W is the width (small diameter) and L is the length (large diameter) of the tumor measured in centimeters. Mouse care and experimental procedures were performed according to the guidelines of the Animal Care Committee of the Taipei Medical University, Taiwan.

### ELISA

Anti-CSE1L (H2) antibody-coated 96-well plates (Costar) were blocked with 5% BSA in TBS (Tris-buffered saline) for 1 h. The wells were washed with TBST (0.05% Tween 20 in TBS) and then incubated with serum samples (sixfold dilution with TBS) for 1 h. After being washed with TBST, the wells were incubated with biotin-conjugated anti-phospho-CSE1L antibodies for 1 h. The biotin-conjugated anti-phospho-CSE1L antibodies were prepared by biotinylating anti-phospho-CSE1L antibodies using the Biotin Labeling Kit-NH2 (Dojindo Laboratories, Kumamoto, Japan). The wells were then washed with TBST, reacted with streptavidin-conjugated horseradish peroxidase (R&D Systems, Minneapolis, MN, USA), and followed by incubation with the substrate reagent (R&D Systems). For calibration, two blank wells containing TBST were used as the background value, and two wells that were not coated with anti-phospho-CSE1L antibodies but reacted with all other ELISA reagents were used as control wells. The absorbance at 450 nm was measured within 20 min following the reaction by using a Thermo Multiskan EX microplate photometer (Thermo Fisher Scientific, Waltham, MA, USA). Each sample was assayed two times.

### Statistical analysis

Statistical differences were analyzed using paired *t* tests. An α level of 0.05 was used to determine statistical significance.

## Results

### Presence of hyper-, hypo-, and non-phosphorylated CSE1L in tumor cells as analyzed by antibodies against CSE1L and phosphorylated CSE1L

**Figure 1:**
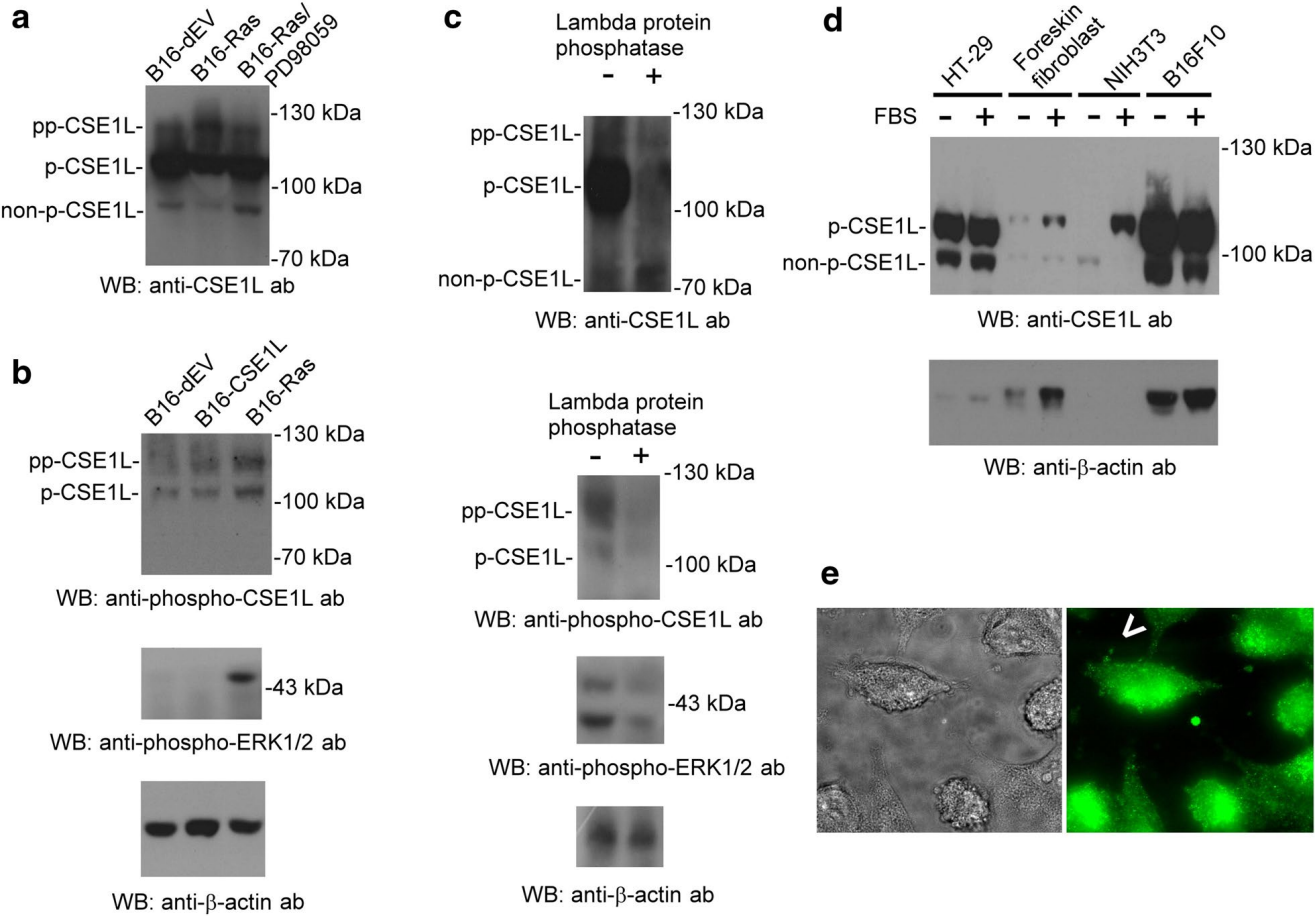
Presence of hyper-, hypo-, and non-phosphorylated CSE1L in tumor cells as analyzed by antibodies against CSE1L and phosphorylated CSE1L. **a** The levels of phosphorylated and non-phosphorylated CSE1L in B16-dEV, B16-Ras, and PD98059-treated B16-Ras cells were analyzed with anti-CSE1L antibody (clone 3D8) as indicated. **b** The levels of phospho-CSE1L and phospho-ERK1/2 in B16-dEV, B16-CSE1L, and B16-Ras cells were analyzed with anti-phospho-CSE1L and anti-phospho-ERK1/2 antibodies as indicated. **c** Presence of hyper- and hypo-phosphorylated CSE1L in B16-Ras cells analyzed using lambda protein phosphatase. B16-Ras cell lysates treated with or without lambda protein phosphatase were subjected to immunoblotting with anti-CSE1L (3D8), anti-phospho-CSE1L, and anti-phospho-ERK1/2 antibodies as indicated. **d** Presence of non-phosphorylated and phosphorylated CSE1L as analyzed using serum-starved and serum re-fed non-cancerous cell lines. The phosphorylation of CSE1L in cell lysates from serum starved or serum starved and serum retreated HT-29 colorectal cancer cells, human foreskin fibroblast cells, NIH3T3 cells, and B16F10 melanoma cells were analyzed using the anti-CSE1L (clone 24) antibody. **e** Distribution of secretory phospho-CSE1L in the extracellular secretion vesicles (*arrowhead*) surrounding B16-Ras cells was analyzed by immunofluorescence with anti-phospho-CSE1L antibodies. Each immunoblot was repeated at least three times and showed similar results. The data shown here are the representative immunoblots. β-actin levels were assayed as a control. *pp-CSE1L* hyper-phosphorylated CSE1L, *p-CSE1L* hypo-phosphorylated CSE1L, *non-p-CSE1L* non-phosphorylated CSE1L.

The results of immunoblotting with the anti-CSE1L antibodies (clone 3D8) showed that there were three CSE1L protein bands with molecular weights of approximately 115, 100, and 90 kDa, and the anti-CSE1L antibodies mainly recognized the 100-kDa CSE1L protein (Figure 1a). The B16-Ras cells exhibited an increased 115-kDa CSE1L level and decreased 100 and 90-kDa CSE1L levels compared with that of B16-dEV cells (Figure 1a). PD98059 is an ERK1/2 activity inhibitor, and PD98059-treated B16-Ras cells exhibited a decreased 115-kDa CSE1L level and increased 100 and 90-kDa CSE1L levels compared with that of B16-Ras cells (Figure 1a). There is no commercial antibody against phospho-CSE1L for detecting phosphorylated CSE1L thus far. Therefore, we developed antibodies specific to phospho-CSE1L. Immunoblotting showed that Ras activation resulted in increased expression of phospho-ERK1/2 (Figure 1b). Immunoblotting with cell lysates from B16-dEV cells and B16-Ras cells showed that the anti-phospho-CSE1L antibodies recognized hypo- and hyper-phosphorylated CSE1L proteins, and Ras activation resulted in increased expression levels of hypo- and hyper-phosphorylated CSE1L in B16-Ras cells (Figure 1b).

We studied the phosphorylation of CSE1L with lambda protein phosphatase which can digest the phosphorylated residue in CSE1L. The results of immunoblotting using lambda-protein-phosphatase-treated cell lysates, anti-CSE1L antibodies (clone 3D8), and anti-phospho-CSE1L antibodies indicated that the 115-kDa protein was hyper-phosphorylated CSE1L, the 100-kDa protein was hypo-phosphorylated CSE1L, and the 90-kDa protein was non-phosphorylated CSE1L (Figure 1c). In addition, lambda protein phosphatase treatment resulted in decreased expression of phospho-ERK1/2 (Figure 1c). These results indicated that tumor cells express hyper-, hypo-, and non-phosphorylated CSE1L, and the anti-phospho-CSE1L antibodies recognize hyper- and hypo-phosphorylated CSE1L but not recognize non-phosphorylated CSE1L.

Immunoblotting with anti-CSE1L antibodies (clone 24) showed that hypo-phosphorylated CSE1L was highly expressed in HT-29 colorectal cancer cells and B16F10 melanoma cells and was nearly undetectable in human foreskin fibroblast cells and NIH 3T3 mouse embryo fibroblast cells cultured under serum starvation (Figure 1d). Serum re-supplement resulted in increased hypo-phosphorylated CSE1L levels and decreased non-phosphorylated CSE1L levels in NIH 3T3 cells (Figure 1d). These results confirmed that the 100-kDa band was hypo-phosphorylated CSE1L and the 90-kDa band was non-phosphorylated CSE1L and indicated that anti-CSE1L antibodies mainly recognized hypo- and non-phosphorylated CSE1L.

The results of immunofluorescence using anti-phospho-CSE1L antibodies showed that phospho-CSE1L was mainly distributed in the cytoplasm of B16F10 cells (Figure 1e). In addition, the results showed the presence of secretory phospho-CSE1L in the extracellular vesicles surrounding B16-Ras cells (Figure 1e). The results indicated that phospho-CSE1L is a secretory protein.

### Vemurafenib and sorafenib treatment inhibited the phosphorylation of CSE1L and ERK1/2

**Figure 2:**
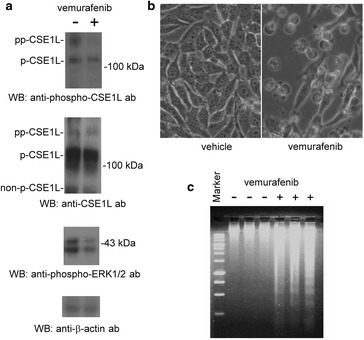
Vemurafenib treatment inhibits the phosphorylation of CSE1L and ERK1/2. **a** Vemurafenib treatment inhibited the phosphorylation of ERK1/2 and CSE1L. The levels of hyper-phosphorylated CSE1L, hypo-phosphorylated CSE1L, and phospho-ERK1/2 in A375 melanoma cells treated with or without 1 μM vemurafenib for 24 h were subjected to immunoblotting with anti-CSE1L (clone 3D8), anti-phospho-CSE1L, and anti-phospho-ERK1/2 antibodies. β-actin levels were assayed as a control. **b** A representative image shows vemurafenib-induced apoptotic body formation in A375 melanoma cells. Cells were treated with or without 1 μM vemurafenib for 72 h. **c** DNA fragmentation induced by vemurafenib in A375 melanoma cells treated with or without 1 μM vemurafenib for 72 h. Each immunoblot was repeated at least three times and showed similar results. The data shown here are the representative immunoblots.

We studied the effects of vemurafenib and sorafenib on the phosphorylation of CSE1L. A375 melanoma cells were treated with 1 μM vemurafenib for 24 h. The results of immunoblotting with anti-phospho-CSE1L antibodies showed that vemurafenib treatment, compared with treatment with the vehicle solution (DMSO), reduced the hyper-phosphorylated CSE1L level and inhibited the phosphorylation of ERK1/2 in A375 cells (Figure 2a). Although vemurafenib treatment reduced hypo-phosphorylated CSE1L levels, the decrease was not significant. Because the anti-CSE1L antibodies reacted more strongly with hypo-phosphorylated CSE1L than with hyper-phosphorylated CSE1L, immunoblotting with the anti-CSE1L antibodies showed that vemurafenib treatment did not significantly reduce the total CSE1L level (Figure 2a). Thus, anti-phospho-CSE1L antibody was much more useful than anti-CSE1L antibody in determining the variation in the phosphorylation status of CSE1L induced by vemurafenib (Figure 2a). Vemurafenib treatment also induced apoptotic body formation and DNA fragmentation in A375 melanoma cells in a longer treatment time (72 h) (Figure 2b, c). Since the phosphorylation of CSE1L is regulated by ERK1/2, these results indicated that vemurafenib inhibited ERK1/2 phosphorylation and reduced the level of CSE1L phosphorylation.

**Figure 3:**
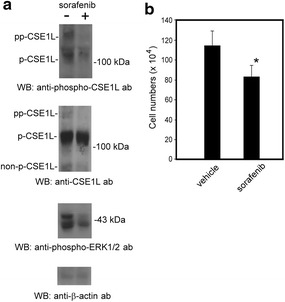
Sorafenib treatment inhibits the phosphorylation of CSE1L and ERK1/2. **a** Sorafenib treatment inhibited the phosphorylation of ERK1/2 and CSE1L. The levels of hyper-phosphorylated CSE1L, hypo-phosphorylated CSE1L, and phospho-ERK1/2 in HT-29 colorectal cancer cells treated with or without 6 μM sorafenib for 24 h were subjected to immunoblotting with anti-CSE1L (clone 3D8), anti-phospho-CSE1L, and anti-phospho-ERK1/2 antibodies. β-actin levels were assayed as a control. Each immunoblot was repeated at least three times and showed similar results. The data shown here are the representative immunoblots. **b** The cell numbers of HT-29 colorectal cancer cells treated with or without 6 μM sorafenib for 96 h were counted using trypan blue exclusion assays. The *graph* summarizes the results of three independent assays.

HT-29 colorectal cancer cells were treated with 6 μM sorafenib for 24 h. The results of immunoblotting showed that sorafenib treatment inhibited ERK1/2 phosphorylation in HT-29 cells (Figure 3a). The results of immunoblotting with anti-phospho-CSE1L antibodies showed that sorafenib treatment resulted in the decline in the hyper- and hypo-phosphorylated CSE1L levels (Figure 3a). The results of immunoblotting showed that anti-phospho-CSE1L antibody was more useful than anti-CSE1L antibody in determining the variation in the phosphorylation status of CSE1L induced by sorafenib (Figure 3a). Sorafenib treatment also inhibited the proliferation of HT-29 cells by 30% in a longer treatment time (96 h) (Figure 3b).

### Serum phospho-CSE1L level declined following vemurafenib and sunitinib treatment in mice inoculated with human melanoma xenografts

**Figure 4:**
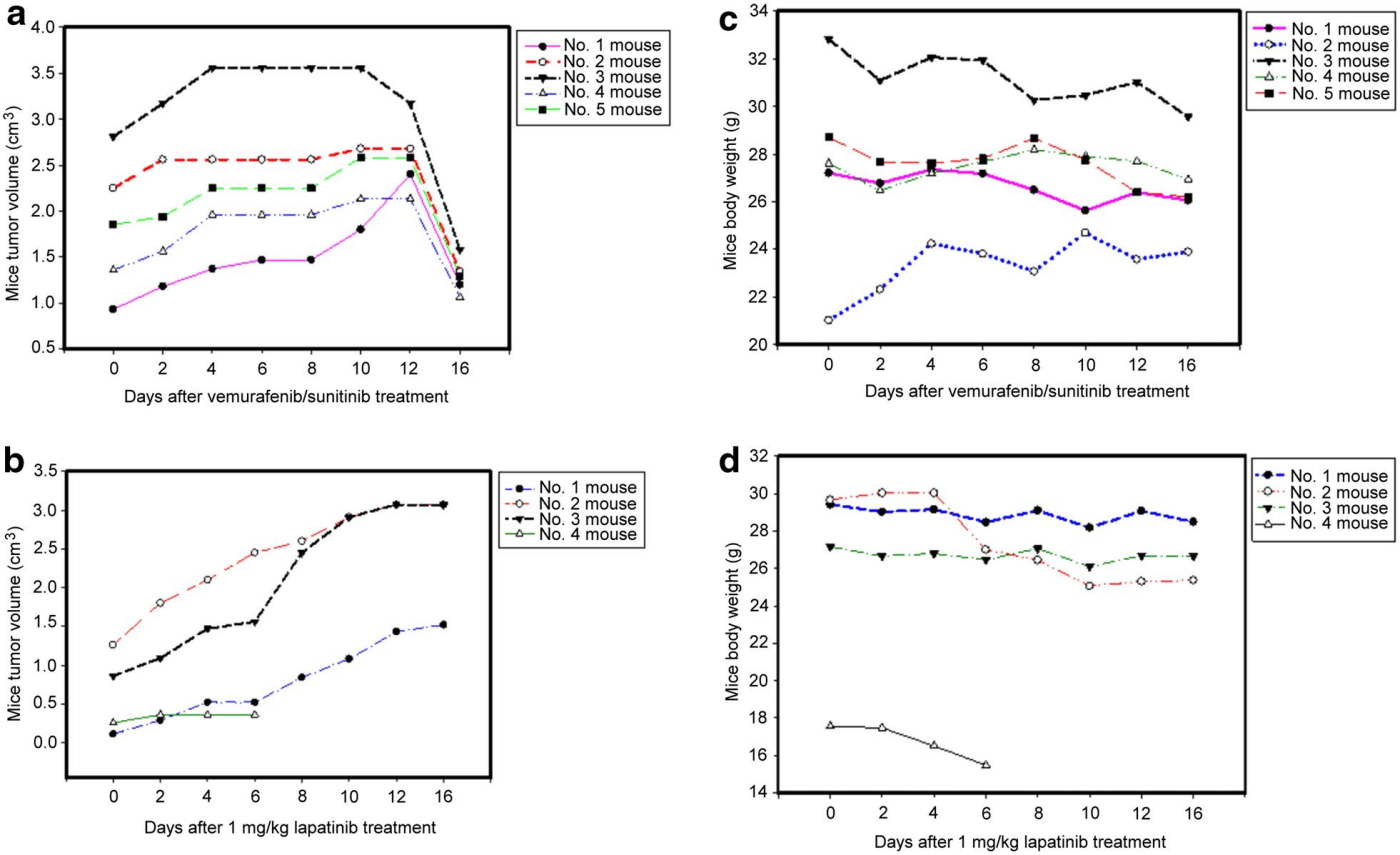
Vemurafenib and sunitinib treatment inhibits tumor growth in mice inoculated with human melanoma xenografts. Male NOD SCID mice bearing tumors derived from human A375 melanoma cells were fed daily with 75 mg/kg of vemurafenib and 20 mg/kg of sunitinib (**a**, **c**) or 1 mg/kg of lapatinib, which was used as the control (**b**, **d**), for 10 days. The tumor size (**a**, **b**) and body weights (**c**, **d**) of each mouse were measured every 2 days as indicated.

**Figure 5:**
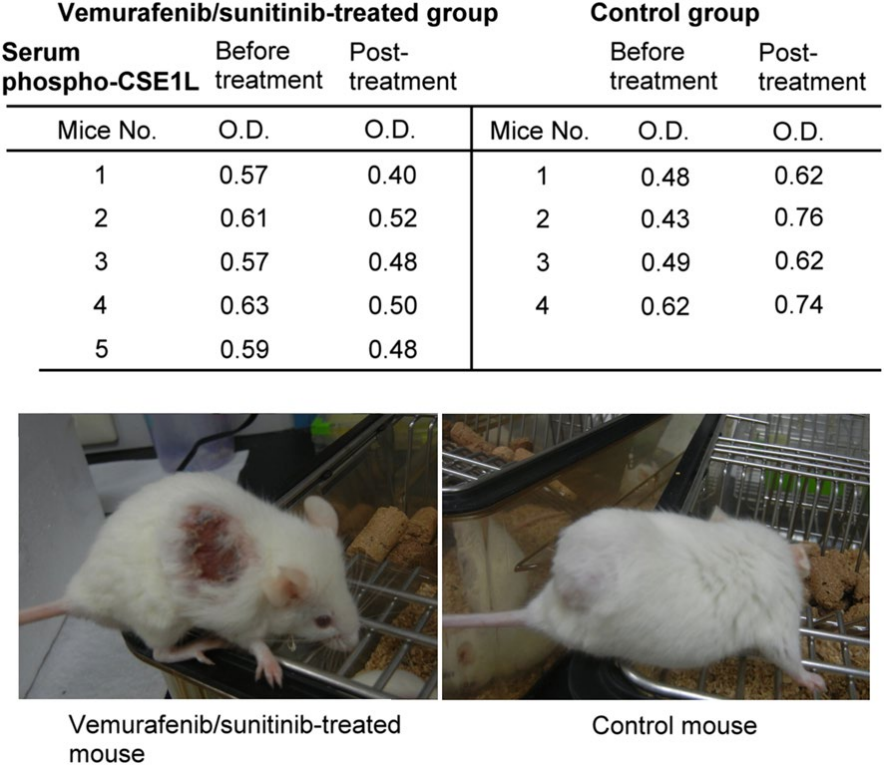
Serum phospho-CSE1L levels decline after vemurafenib and sunitinib treatment in mice inoculated with human melanoma xenografts. ELISA analysis of phospho-CSE1L levels in serum samples obtained from NOD SCID mice bearing human A375 melanoma xenografts and fed daily with 75 mg/kg of vemurafenib plus 20 mg/kg of sunitinib or 1 mg/kg of lapatinib as the control for 10 days. Sera were collected from mice 1 day prior to drug administration and 5 days following the onset of drug administration. The ELISA assays were performed two times and showed similar results. The data shown here are representative results. The images in the lower panel are representative images of a mouse treated with vemurafenib/sunitinib and showing tumor necrosis and a mouse from the control group 21 days after drug administration. *O.D*. optical density.

We used mice tumor xenograft model to study the assay of serum phospho-CSE1L for detecting the efficacy of targeted drugs. NOD SCID mice bearing tumors derived from A375 human melanoma cells were fed daily with 75 mg/kg of vemurafenib and 20 mg/kg of sunitinib or an ineffective prescription of targeted treatment, 1 mg/kg of lapatinib, for 10 days. The results showed that vemurafenib and sunitinib treatment significantly inhibited the growth of melanoma xenografts derived from A375 cells, even when treatment was initiated until the tumors were quite large. On the basis of caliper measurements, inhibition of tumor proliferation was observed on Day 6 after vemurafenib and sunitinib treatment (Figure 4a). Significant tumor regression (large area of tumor necrosis) was observed on Day 16 after vemurafenib and sunitinib treatment in all five mice (Figures 4a, 5).

In the control, mice bearing established A375 melanoma were fed with 1 mg/kg of lapatinib daily for 10 days. This study design mimics the clinical situation of targeted therapy in which a prescription of a targeted drug or the dose of a drug may be incorrect. Lapatinib treatment was inefficacious against melanoma xenografts derived from A375 melanoma cells (Figure 4b). Neither tumor stasis (i.e. inhibition of additional tumor growth) nor reduction of tumor volume occurred after lapatinib treatment in all four mice (five mice were used in the control group, one mouse died and was excluded from the study) throughout the study period (Figure 4b).

The body weights of mice treated with vemurafenib and sunitinib were measured and observed to decrease by approximately 1 g in two mice, 2 g in one mouse, and 3 g in one mouse, whereas the body weight of one mouse increased by approximately 2.5 g (Figure 4c). In mice treated with 1 mg/kg of lapatinib, the body weights decreased by approximately 1 g in two mice and 2 g in two mice (Figure 4d). Because mice treated with 1 mg/kg of lapatinib also exhibited a decrease in body weight, the decrease in the body weights of the mice treated with vemurafenib and sunitinib may not have been because of drug toxicity. The decrease may have resulted from exsanguinations in the experimental procedure.

In addition, the assay results and comparison of blood samples collected from mice a day prior to targeted drug treatment and 5 days after targeted drug treatment by ELISA method showed that serum phospho-CSE1L levels declined 5 days after vemurafenib and sunitinib treatment in all five mice, whereas serum phospho-CSE1L levels increased 5 days following 1 mg/kg of lapatinib treatment in all four mice bearing A375 melanoma xenografts (Figure 5). The optical density (O.D.) values of serum phospho-CSE1L levels 1 day prior and 5 days after vemurafenib/sunitinib treatment and lapatinib treatment are shown in the table (Figure 5).

### Serum phospho-CSE1L levels declined after sorafenib and lapatinib treatment in mice inoculated with human colorectal tumor xenografts

**Figure 6:**
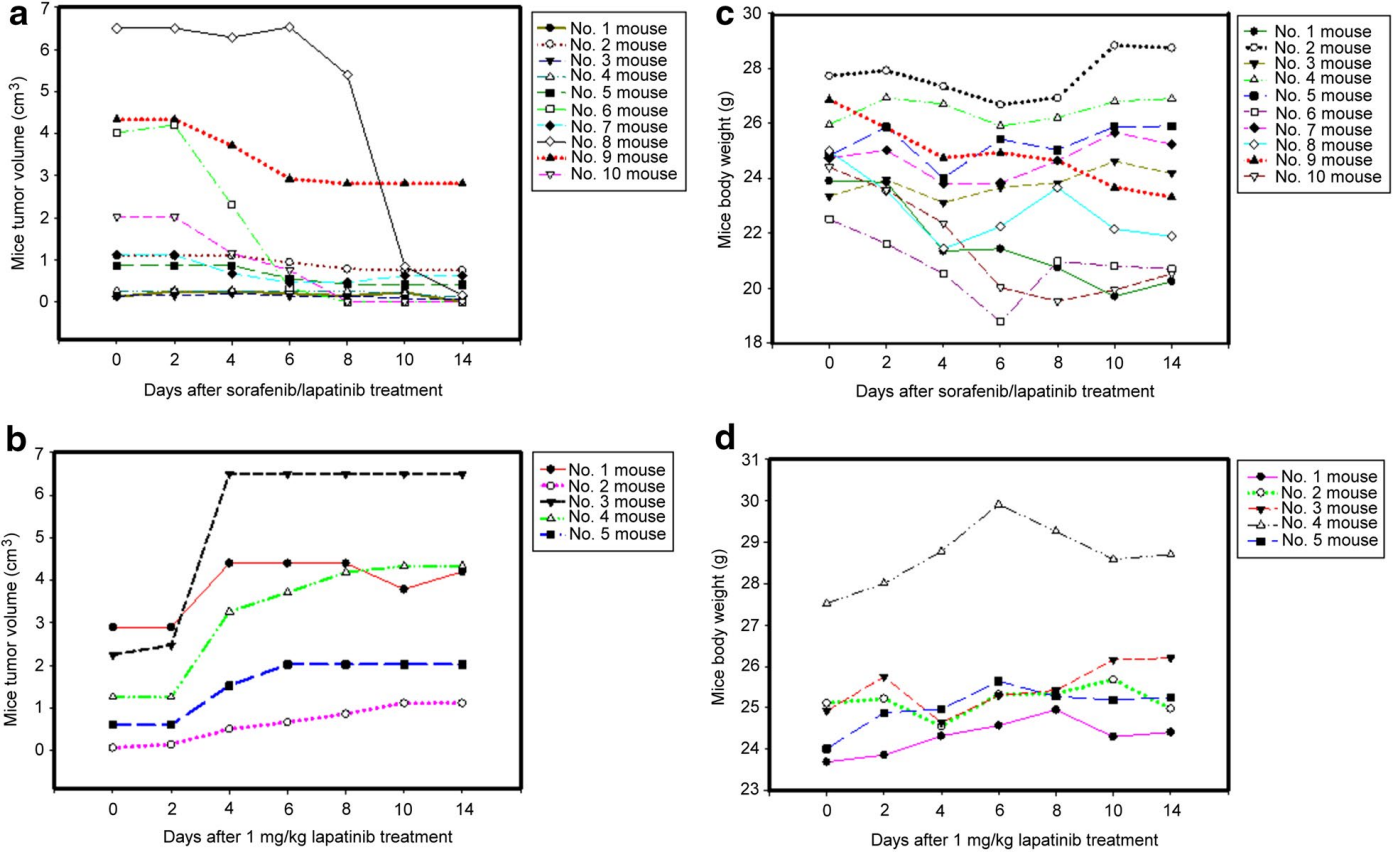
Sorafenib and lapatinib treatment inhibits tumor growth in mice inoculated with human colorectal tumor xenografts. Male NOD SCID mice bearing tumors derived from human HT-29 colorectal cancer cells were fed daily with 80 mg/kg of sorafenib and 60 mg/kg of lapatinib (**a**, **c)** or 1 mg/kg of lapatinib as the control (**b**, **d**) for 10 days. The tumor size (**a**, **b**) and body weights (**c**, **d**) of each mouse were measured every 2 days as indicated.

**Figure 7:**
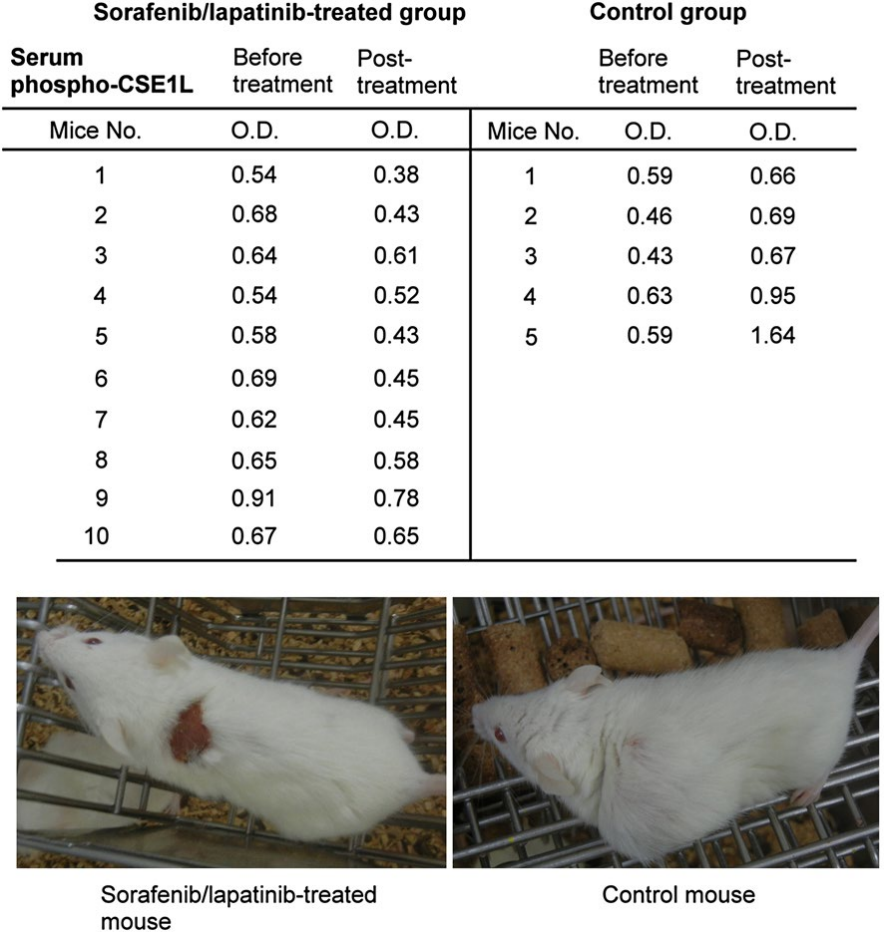
Serum phospho-CSE1L levels decline after sorafenib and lapatinib treatment in mice inoculated with human colorectal tumor xenografts. ELISA analysis of phospho-CSE1L levels in serum samples obtained from NOD SCID mice bearing HT-29 colorectal tumor xenografts and fed daily with 80 mg/kg of sorafenib plus 60 mg/kg of lapatinib or 1 mg/kg of lapatinib as the control for 10 days. Sera were collected from the mice 1 day prior to drug administration and 3 days after the first drug administration. The ELISA assays were performed two times and showed similar results. The data shown here are the representative results. The images in the *lower panel* are representative images of a mouse treated with sorafenib/lapatinib and showing tumor necrosis and a mouse from the control group 21 days after drug administration.

The assay of serum phospho-CSE1L for detecting the efficacy of targeted treatment was also conducted in the HT-29 human colorectal tumor xenograft model. Mice bearing established HT-29 colorectal tumors were administered oral doses of 80 mg/kg of sorafenib and 60 mg/kg of lapatinib daily for 10 days. The doses of sorafenib and lapatinib were effective and the treatment significantly inhibited the growth of tumor xenografts derived from HT-29 cells in all 10 mice, even when drug treatment was initiated until the tumors were quite large. Decrease in tumor volume was observed on Day 4 after sorafenib and lapatinib treatment (Figure 6a). Complete tumor regression (complete tumor necrosis) occurred on Day 14 after sorafenib and lapatinib treatment in 3 of 10 mice (Figures 6a, 7).

For control treatment, mice bearing established HT-29 colorectal tumors were fed with 1 mg/kg of lapatinib daily for 10 days. Lapatinib was inefficacious against tumor xenografts derived from HT-29 colorectal cancer cells (Figure 6b). Caliper measurements of the tumors indicated that lapatinib treatment resulted in neither inhibition of additional tumor growth nor reduction in tumor size in all five mice throughout the study period (Figure 6b).

Following sorafenib and lapatinib treatment, the body weights decreased by approximately 2–3 g in four mice and 4 g in one mouse, and no such loss in body weight was observed in five mice (Figure 6c). In mice treated with lapatinib, the body weight decreased by approximately 0.2 g in one mouse, and no such decrease occurred in four mice (Figure 6d). Because the decrease in body weight was also observed in the control mice, the decreased body weight of mice treated with sorafenib and lapatinib may not have been because of drug toxicity. The decrease in body weight may have resulted from exsanguinations in the study.

The assay of serum phospho-CSE1L levels in the blood samples collected from mice 1 day prior and 3 days after targeted drug treatment by ELISA method indicated that serum phospho-CSE1L levels declined 3 days after drug treatment in all 10 mice fed daily with 80 mg/kg of sorafenib and 60 mg/kg of lapatinib (Figure 7). The O.D. values of serum phospho-CSE1L levels 1 day prior and 3 days after sorafenib and lapatinib treatment are shown in the table (Figure 7). In mice fed daily with 1 mg/kg of lapatinib, the serum phospho-CSE1L levels increased 3 days after drug treatment in all five mice inoculated with HT-29 colorectal tumor xenografts (Figure 7). The O.D. values of serum phospho-CSE1L levels 1 day prior and 3 days after 1 mg/kg lapatinib treatment are shown in the table (Figure 7).

## Discussion

Targeted therapy can substantially improve clinical outcomes of cancer treatment and has provided cancer patients with new hope. However, numerous patients continue to experience tumor resistance to a targeted drug in initial treatment or after successful initial therapy [1–3, 38]. When the development of resistance to a targeted drug is detected, tumor often has progressed; this leads to disease treatment more difficult and ultimately causing death. Detecting the change in the tumor size according to PET/CT (positron emission tomography-computed tomography) scans is the main method for monitoring targeted therapy 2–3 months after drug treatment. PET/CT using fluorine-18-fluorodeoxyglucose (FDG-PET/CT) can be used to evaluate glucose metabolism changes in tumors as early as 1 week after targeted drug treatment in some cancer types and some kinds of targeted drugs, and it plays a role in defining tumor response to targeted therapy in tumors such as malignant lymphoma and gastrointestinal stromal tumors treated with imatinib [39, 40]. However, false-positive or equivocal results are a known limitation of FDG-PET/CT in cancer evaluation [41]. The metabolism of glucose is mediated by Akt (v-Akt murine thymoma viral oncogene)/PKB (protein kinase-B) pathway, and Akt is regulated by EGFR-PI3K (phosphatidylinositol-3-kinase) signaling [42]. Accordingly, it should not be assumed that FDG-PET/CT can be used for assaying the therapeutic response of all molecular-targeted therapies. Therefore, using FDG-PET/CT for monitoring the tumor response to targeted therapy in all cancer types and all cancer patients is difficult. Gebhart et al. reported that, in 86 HER2-positive breast cancer patients, only 77 patients had an evaluable baseline signaling using (18)F-FDG PET/CT scans [43]. FDG-PET/CT cannot reliably detect tumors <0.5 cm in size and, thus, cannot reliably detect emerging metastasis in tumors that acquire resistance to targeted drugs and undergoing metastasis [44, 45]. Timely monitoring of the onset of disease recurrence or the emergence of secondary resistance to targeted drugs continues to be a major challenge. Our previous study showed that the phosphorylation of CSE1L is regulated by ERK1/2 signaling, an essential signaling downstream of most targets in cancer cells in targeted therapy [4–7, 31]. Our present results indicated that phosphorylated CSE1L is a secretory protein (Figure 1); vemurafenib and sorafenib treatment inhibited ERK1/2 and CSE1L phosphorylation (Figures 2, 3); and serum phospho-CSE1L levels declined 3–5 days after the administration of targeted drug therapies, namely vemurafenib and sunitinib treatment and sorafenib and lapatinib treatment, in tumor xenograft models (Figures 4, 5, 6, 7). These results indicated that serum phospho-CSE1L is a potential marker for the early detection of the efficacy of targeted therapy.

CSE1L is highly expressed in most cancer types and it is a secretory protein [25–30]. By using 2D gel electrophoresis, Scherf et al. reported the presence of the cellular phosphorylated and non-phosphorylated forms of CSE1L [46]. Our previous study showed that healthy donor serum samples also contain certain levels of CSE1L, thus although the serum CSE1L level of cancer serum samples was slightly higher than that of healthy donor serum samples, the difference was not as significant as that observed in an assay of phosphorylated CSE1L [31]. Since CSE1L is also secreted from the normal cells in cancer patients, assay of serum phospho-CSE1L is more effective than that using serum CSE1L for cancer diagnosis as well as for the early detection of the efficacy of targeted therapy in patients.

Monitoring emerging secondary drug resistance is a crucial clinical setting in targeted cancer therapy because certain tumors develop resistance to targeted drugs after successful initial treatment. For example, tumors develop resistance to vemurafenib through the following mechanisms: tumor cells begin to overexpress PDGFR-B [47], *N*-*ras* mutation reactivates the normal B-Raf pathway [47], tumors induce feedback activation of the EGF receptor [48], and stromal cells around tumor secrete hepatocyte growth factor [49]. All of these alternatively activated growth factor receptors and kinases can reactivate MEK-ERK signaling in vemurafenib-treated (resistance) tumor cells, and will result in CSE1L phosphorylation. Thus, phospho-CSE1L is a potential biomarker for monitoring the emergence of secondary drug resistance after successful initial targeted therapy, at least for detecting tumor resistance to vemurafenib.

Although targeted drugs are more specific to tumors and do not affect the body in the same way that standard chemotherapeutic drugs do, targeted cancer drugs still cause side effects. For example, tumor blood vessel angiogenesis inhibitors affect the angiogenesis of not only vessels located near the tumor but also those in other organs [50]. Thus, because of the side effects of targeted drugs, the drug dose may need to be reduced in some clinical situations. In our study, we used an ineffective targeted treatment, i.e. 1 mg/kg lapatinib, as the control treatment, and the results indicated that this treatment neither caused tumor regression nor reduced serum phospho-CSE1L levels in the mice tumor xenograft model (Figures 4, 5, 6, 7). Thus, in addition to enabling early detection of the efficacy of a targeted drug in initial treatment and monitoring of the development of resistance to targeted therapy after successful initial treatment, assay of serum phospho-CSE1L may provide insight into the effect of dose reduction in the clinical setting and, thus, may have clinical utility in monitoring the optimal dose of a drug in targeted therapy (or combined therapy with targeted and chemotherapeutic drugs).

## Conclusions

Our findings suggest that serum phospho-CSE1L has clinical utility in the early detection of the efficacy of targeted therapy as well as monitoring emerging secondary drug resistance in cancer patients, thus facilitating timely treatment decision making.
